# Associations of parental attitudes and health behaviors with children’s screen time over four years

**DOI:** 10.1186/s12889-023-15212-x

**Published:** 2023-02-08

**Authors:** Qian-Wen Xie, Roujia Chen, Xudong Zhou

**Affiliations:** 1 Department of Social Welfare and Risk Management, School of Public Affairs, Zhejiang University, Zijin’ gang Campus, 866 Yuhangtang Rd, 310058 Hangzhou, China; 2 Research Center for Common Prosperity, Future Regional Development Laboratory, Innovation Center of Yangtze River Delta, Zhejiang University, Jiaxing, China; 3 Center of Social Welfare and Governance, Zhejiang University, Hangzhou, China; 4 The Institute of Social and Family Medicine, School of Medicine, Zhejiang University, 866 Yuhangtang Rd, 310058 Hangzhou, China; 5 The Second Affiliated Hospital, Zhejiang University School of Medicine, Hangzhou, China

**Keywords:** Screen time, Physical activity, Children, Parental attitude, Health behavior, Healthy family climate, Cigarette smoking, Alcohol drinking

## Abstract

**Background:**

Parents are significantly important in shaping the screen use of children within a family system. This study aimed to examine the associations of Chinese children’s screen time (ST) over four years with parents’ attitudes toward their own screen use and physical activities (PA) and health behaviors including their ST, PA, cigarette smoking, and alcohol drinking.

**Methods:**

The current study utilized data from two waves (2011 and 2015) of the China Health and Nutrition Survey (CHNS), including 1,941 mother-father-child triads in 2011 and 2,707 mother-father-child triads in 2015 (with children aged 0-17-years-old). The ST of children and the parental attitudes and health behaviors were measured via self-report or proxy-report (for children under 6 years old) questionnaires. Pool-OLS regression models were used to assess the associations of parental attitudes and health behaviors with the ST of children. Moderation models were built to assess whether these associations depended on the gender, age, and family income of children, as well as whether paternal and maternal influences were moderated by the other parent. A multilevel cross-lagged panel model (CLPM) was used to assess parental influences on children’s ST over four years.

**Results:**

Paternal ST (β = 0.09, p < 0.001), maternal ST (β = 0.10, p < 0.001), and paternal alcohol drinking (β = 0.30, p < 0.05) were positively associated with children’s ST. In addition, maternal smoking had a positive association with girls’ ST (β = 0.53, p < 0.05). Moreover, the association between maternal ST and children’s ST was observed to decline as family income increased (β = -0.03, p < 0.001). Paternal ST had a larger positive association with children’s ST when the ST of mothers exceeded 14 h/week (β = 0.06, p < 0.05). Furthermore, lagged associations were found between paternal attitudes toward PA (β = -1.63, p < 0.05) or maternal cigarette smoking (β = 1.46, p < 0.05) and children’s ST measured four years later.

**Conclusion:**

Children establish a healthy lifestyle within the family system. From the perspective of the healthy family climate, the current study suggests that future programs for reducing children’s ST should be built through an integrative approach with special attention to parental attitudes and health behaviors.

**Supplementary Information:**

The online version contains supplementary material available at 10.1186/s12889-023-15212-x.

## Introduction

Excessive screen time (ST) is associated with negative health outcomes across the lifespan, including developmental delays in early childhood [1], obesity in childhood [2], and cardiometabolic risk in adolescence [3]. Furthermore, screen use patterns formed during childhood may progress into adulthood [4]. The American Academy of Pediatrics (AAP) recommends no ST for children under 2 years, no more than 1 h for 2- to 5- years old children, and less than 2 h for children aged 6 years and older [5]. However, the average daily ST for children (5–18 years old) around the world averaged 3.6 h according to a meta-analysis [6]. Thus, it is vital and urgent to help children to establish healthy screen use habits for their short-term and long-term health. The development of healthy lifestyles of children is embedded in a social context, and the family system is likely the most proximal and influential social environment. An aggregation of family members’ attitudes and health behaviors can represent a family’s shared values concerning health, which defines a family health climate [7]. From this perspective, the current study aimed to explore the associations of parental attitudes and health behaviors with children’s ST.

Previous studies have identified parental attitudes as important determinants of children’s ST. Evidence has shown that parents’ negative outcome expectations regarding children’s use of screens [8], positive perceptions toward limiting children’s ST, and more knowledge of official ST recommendations for children [9] are associated with less children’s ST. However, most of these studies concentrated on parental attitudes toward their children’s screen use rather than their own health behaviors. From the perspective of the family health climate, the specific motives, affects, and behaviors of all of the family members influence each other within a family system [7]. In fact, research on other lifestyles has discovered that parental motivation for engaging in physical activity (PA) served as an important construct that impacted children’s PA [10]. Yet, it remains unexplored as to whether parental attitudes toward their own screen use and other lifestyles are associated with children’s ST.

With regard to parental health behaviors, the importance of parents as “role models” in shaping children’s screen use has been widely recognized in previous studies. A consistent positive relationship between parental and children’s ST was found in both cross-sectional and longitudinal studies [11, 12]. Nevertheless, most of these studies have ignored the potential clustering effect of parental health behaviors. According to the health lifestyle theory [13], the healthy or unhealthy behaviors of individuals tend to exhibit a collective pattern because the choices of health-related behaviors mainly depend upon the available options and the value judgments about them, which are both defined by a person’s social position. In accordance with this theory, the clustering of excessive ST, physical inactivity, overuse of cigarettes, and overuse of alcohol was observed at the intrapersonal level [14, 15]. A cluster of parental health behaviors has been considered to represent the general value that a household places on healthy living [16], which is also an important component of the family health climate [7]. To the best of our knowledge, no study has examined the associations between parents’ PA or substance use behaviors and children’s ST.

Moreover, parental influences are more than the sum of father-child and mother-child associations within a family system, as the family health climate is also developed in the process of family members’ interrelationships [7]. Children live in a mother-father-child triadic system, wherein father-child relationships should be studied in the context of mother-child relationships and vice versa [17, 18]. Although previous research has agreed on the strong father-child and mother-child associations in ST [16, 19], it is unclear as to whether these associations would depend on the other parent. In addition, children’s ST has also been associated with sociodemographic factors, such as gender, age, and family income [20]. The issue as to whether parental influences on children’s ST differ according to these factors is still poorly understood.

Therefore, the current study aimed to address these gaps in the literature by examining (1) the associations of parental attitudes toward their own health behaviors, such as television (TV) viewing and PA, with children’s ST; (2) the associations between parental ST, PA, cigarette smoking, and alcohol drinking and children’s ST; (3) whether these associations are moderated by the gender, age, and family income of children; (4) whether paternal influences on children’s ST are moderated by mothers’ health behaviors and vice versa; and (5) the lagged associations of parental attitudes and health behaviors with children’s ST at a later time point.

## Methodology

### Participants

The current study utilized data from the most recent two waves (2011 and 2015) of the China Health and Nutrition Survey (CHNS), which is a longitudinal household-based study with ten waves ranging from 1989 to 2015. The CHNS sample encompasses individuals from 12 provinces and municipalities in different regions in China. A multistage random cluster sample design was adopted in each province. First, counties in each province were stratified by average income status (low-, middle-, and high-income). Five counties (one high-income, two middle-income, and two low-income counties) were randomly selected using a weighted sampling scheme, in addition to the provincial capital city (if feasible). Afterward, one township and three villages from each county or two urban and two suburban communities from each municipality were randomly selected. Community-, household-, and individual-level surveys were conducted by trained field staff in each wave. The survey was approved by the Institutional Review Boards of the University of North Carolina at Chapel Hill and the National Institute of Nutrition and Food Safety, China Center for Disease Control and Prevention. In the current study, children under the age of 18 years and whose parents both participated in the CHNS were sampled. There were 1,941 mother-father-child triads in 2011 and 2,707 mother-father-child triads in 2015 that were included in the study. Out of the entire sample, 1,014 mother-father-child triads participated in both years.

### Measures

#### Parental and children’s ST and PA

In the CHNS, recreational ST was measured, including watching TV/videotapes, playing video games, playing smartphones, online chatting, and surfing the internet. PA included walking, running, swimming, dancing, ball sports (e.g., basketball and tennis), and other exercises (e.g., gymnastics or martial arts). All of the participants were asked to answer questions about their weekday and weekend daily time spent on participation in each type of screen use and PA. For those who were under 6 years old, parents or other guardians answered questions on their behalf. Total hours per week were computed with [5*(weekday ST/PA hours) + 2*(weekend ST/PA hours)] as the measurement of ST and PA in the current study. We dichotomized parental ST based on a 14-hour/week threshold according to the suggestion of the National Heart, Lung, and Blood Institute (NHLBI) of the United States [21]. Similar to the previous studies based on the CHNS data [22], parental PA participation was low in the current study (65% of parents had zero participation in PA). Referring to their operationalization, parental PA was dichotomized into any participation versus no participation.

#### Parental attitudes toward their health behaviors

Parental attitudes toward their health behaviors were assessed via two questions. One question concerned parents’ attitudes toward TV viewing. Specifically, both parents were asked “Do you like watching TV?”, which was measured on a five-point Likert-type scale anchored with “1 - Extremely dislike” and “5 - Extremely like”, with higher scores representing greater preference. The other question, “How much do you care about your engagement in physical activity?”, was used to assess parental attitudes toward their PA. This item was based on a five-point Likert-type scale, with “1- Never care” and “5 - Always care”. Dichotomous variables for parental attitudes were developed for the analysis of paternal or maternal moderation effects. Parents who answered “like” and “extremely like” were categorized as having a “high TV watching preference” versus a “low TV watching preference”. Those participants who answered “mostly” and “always” caring about PA were marked as being “conscious about PA” versus “negligent about PA”.

#### Parental cigarette smoking and alcohol drinking

Parents were asked about their history of smoking cigarettes or drinking alcohol. In the current study, the number of cigarettes that parents smoked per day (0 if never smoked) and the frequency of drinking (0 if never drank) were used to measure parental cigarette smoking and alcohol drinking behaviors. Similar dichotomous variables were created referring to previous CHNS studies. “Ever smokers” were defined if the participants had ever smoked [23]. Those participants who reported drinking more than twice a week were identified as being “frequent drinkers” [24].

#### Covariates

Sociodemographic variables were collected in both waves and used as covariates. Children’s characteristics included age, gender, ethnicity (Han/Minority), geographic region (Central/East/Northeast/West), and residence (rural/urban). Parental and family characteristics included parental education level (primary or under/middle school/high school/technical school/college or above), parental employment status (unemployment/fixed-term workers/others), and inflation-adjusted family income per capita.

### Statistical analyses

All of the analyses were conducted in R Studio version 1.3.1093 (RStudio Inc., Boston, MA), and p < 0.05 was used as the significance level. Regarding the missing values, 3–11% of the observations contained missing values in independent, dependent, and covariate variables in both the 2011 and 2015 survey data. Missing data were assumed to be missing at random; therefore, a multiple imputation (MI) was conducted. T tests were performed thereafter to check the mean differences before and after MI, and they were not statistically significant. Descriptive analyses were conducted on both cohorts, which included sample counting and percentages of discrete variables, mean values, and standard deviations of continuous variables. Chi-square and F tests were performed to test the difference between the two waves.

The associations of parental attitudes and health behaviors with children’s ST were examined by building a pooled OLS regression model based on 2011 and 2015 survey data (N = 4,648). The pooled OLS model was chosen to include as many responses as possible, and it has the advantage of considering unbalanced panel data. Assumptions of the pooled OLS (linearity, homoscedasticity, normality of residuals, and no omitted variable) were tested and results are shown in Supplementary Appendix I. To adhere to the assumptions and previous studies on children’s ST [19], children’s age, gender, region, ethnicity, residence, parental education, parental employment status, and survey wave, were used as the control variables in the model. Overall, the model fit the data well (F = 32.24, p < 0.001). To estimate whether the associations of parental attitudes and health behaviors with children’s ST changed as the gender, age, and family income of the children changed, interaction terms were then separately added to the base model. All three models had good model fits (F ≥ 13.00, p < 0.001). To account for the clustering at the family level, confidence intervals (CIs) and p-values were determined using clustered standard errors.

When considering that the associations between children’s ST and one parent’s attitudes and health behaviors may vary depending on those of the other parent, for both mothers and fathers, 6 pooled OLS regression models with moderators (one model for each attitude and behavior) were constructed. In each model, the dependent variable was children’s ST, the independent variable was one maternal/paternal attitude or behavior, and the moderator was the corresponding dichotomous attitude or behavior variable of the other parent. Other parental attitudes and behaviors, as well as the sociodemographics of the parents and children were controlled. All of the 12 models fit the data well (adjusted R^2^ ≥ 0.176; F ≥ 26.47, p < 0.001). Clustered standard errors were used to compute the confidence intervals and p-values.

To examine the lagged associations of parental attitudes and health behaviors with children’s ST at a later time point and consider the clustering at the family level, a multilevel cross-lagged panel model (CLPM) was employed. The multilevel CLPM was based on participants (n = 1,014) who participated in both the 2011 (T0) and 2015 (T1) surveys. By using the CLPM, the lagged variable effect of children’s ST at the previous time point was accounted for. By setting family cluster as one level of the analysis, the interdependence between mothers’ and fathers’ attitudes and behaviors were thus controlled for. The maximum likelihood estimator was used, and goodness of fit was assessed based on the comparative fit index (CFI > 0.95), normed fit index (NFI > 0.95), root mean square error of approximation (RMSEA < 0.05), and standardized root mean square residual (SRMR < 0.08).

## Results

### Descriptive analysis

The descriptive statistics of the key variables are shown in Table 1. The mean ages of children in the 2011 sample were 8.16 years (SD = 5.05) and 8.01 years (SD = 4.74) in the 2015 sample, respectively. The average weekly total ST of children in 2011 was 13.71 h/week (SD = 13.23) and increased (F = 8.06, p < 0.001) to 16.15 h/week (SD = 17.39) in 2015. More than 40% of children’s ST exceeded the recommendations [5] in both 2011 and 2015. Parental weekly total ST also demonstrated an increasing trend from 2011 to 2015 (fathers: F=55.94, p<0.001; mothers: F=33.10, p<0.001). The weekly total time parents spent on PA increased from 2011 to 2015 (fathers: F = 90.44, p < 0.001; mothers: F = 203.12, p < 0.001). When regarding parental attitudes toward their health behaviors, out of a 1–5 scale, parents generally liked watching TV (mean > 3.50) while feeling indifferent about participating in PA (mean ≤ 3.21). There was no obvious difference between paternal and maternal attitudes in either wave (2011: t = 0.29, p = 0.77; 2015: t = -1.25, p = 0.21). When regarding parental smoking and drinking behaviors, fathers consumed far more cigarettes and alcohol than mothers (smoking – 2011: t = 39.62, p < 0.001, 2015: t = 46.59, p < 0.001; alcohol drinking – 2011: t = 40.32, p < 0.001, 2015: t = 48.09, p < 0.001).

**Table 1:** Summary statistics

Wave Variable	2011 (n = 1941)	2015 (n = 2707)	χ^2^ / F
	N (%) / Mean (SD)	N (%) / Mean (SD)	
Children’s ST	13.71 (13.23)	16.15 (17.39)	8.06***
*Parental attitudes and health behaviors*			
Paternal attitude toward TV viewing	3.67 (0.73)	3.50 (0.81)	50.57***
Maternal attitude toward TV viewing	3.70 (0.75)	3.52 (0.84)	54.98***
Paternal attitude toward PA	3.21 (0.78)	3.11 (0.68)	14.32***
Maternal attitude toward PA	3.21 (0.75)	3.14 (0.67)	6.91***
Paternal ST	20.33 (14.47)	24.09 (20.42)	55.94***
Maternal ST	18.33 (12.92)	21.00 (17.76)	33.10***
Paternal PA	1.06 (3.15)	2.19 (3.82)	90.44***
Maternal PA	0.73 (2.48)	2.42 (4.11)	230.12***
Paternal cigarettes smoking	9.92 (10.87)	9.29 (10.27)	1.42
Maternal cigarettes smoking	0.09 (1.16)	0.06 (0.98)	3.04*
Paternal alcohol drinking	3.00 (1.79)	2.82 (1.79)	15.19***
Maternal alcohol drinking	1.23 (0.75)	1.10 (0.51)	52.10***
*Parental and children’s socio-demographics*			
Children’s age	8.16 (5.05)	8.01 (4.74)	0.87
Father’s age	37.73 (7.10)	37.84 (7.03)	0.24
Mother’s age	35.61 (6.73)	35.85 (6.76)	0.17
Children’s gender			1.37
… Boys	1050 (54.1%)	1516 (56.0%)	
… Girls	891 (45.9%)	1191 (44.0%)	
Children’s ethnicity			3.71*
… Minority	199 (10.3%)	316 (11.7%)	
… Han	1742 (89.7%)	2391 (88.3%)	
Region			49.12***
… Central China	472 (24.3%)	841 (31.1%)	
… Eastern China	637 (32.8%)	675 (24.9%)	
… Northeastern China	196 (10.1%)	315 (11.6%)	
… Western China	636 (32.8%)	876 (32.4%)	
Residence			53.39***
… Rural	1174 (60.5%)	1906 (70.4%)	
… Urban	767 (39.5%)	801 (29.6%)	
Paternal education			17.17***
… Primary school or under	316 (16.3%)	348 (12.9%)	
… Middle school	826 (42.6%)	1223 (45.2%)	
… High school	311 (16.0%)	460 (17.0%)	
… Technical school	168 (8.7%)	278 (10.3%)	
… College and above	320 (16.5%)	398 (14.7%)	
Maternal education			38.25***
… Primary school or under	460 (23.7%)	474 (17.5%)	
… Middle school	774 (39.9%)	1281 (47.3%)	
… High school	244 (12.6%)	332 (12.3%)	
… Technical school	156 (8.0%)	211 (7.8%)	
… College or above	307 (15.8%)	409 (15.1%)	
Paternal employment status			74.62***
… Unemployed	218 (11.2%)	581 (21.5%)	
… Fixed-term worker	802 (41.3%)	962 (35.5%)	
… Others	921 (47.4%)	1164 (43.0%)	
Maternal employment status			69.04***
… Unemployed	532 (27.4%)	1104 (40.8%)	
… Fixed-term worker	629 (32.4%)	750 (27.7%)	
… Others	780 (40.2%)	853 (31.5%)	

Significance: *p < 0.05; **p < 0.01; ***p < 0.001;

Measurement units: screen time - hours/week, physical activity - hours/week, cigarette smoking - number of cigarettes smoked per day, alcohol drinking - frequency of drinking during the past week, attitudes toward TV viewing and physical activity − 5-point Likert-scales

### The associations between children’s ST with parental attitudes and health behaviors

The pooled OLS regression model results are shown in Table 2. After controlling for children’s and parental sociodemographics, paternal ST (β = 0.09, 95% CI [0.06, 0.12], p < 0.001), maternal ST (β = 0.10, 95% CI [0.06, 0.13], p < 0.001), and paternal alcohol drinking (β = 0.30, 95% CI [0.05, 0.56], p < 0.05) were positively associated with children’s ST. Parental attitudes toward TV viewing and PA were not significantly associated with children’s ST.

**Table 2:** Pooled OLS regression models of the associations of parental attitudes and health behaviors with the ST of children (N = 4,648)

	Dependent variable: Children’s screen time											
	Base model			Children’s gender moderation			Children’s age moderation			Family income moderation		
	β	95% CI		β	95% CI		β	95% CI		β	95% CI	
		Lower	Upper		Lower	Upper		Lower	Upper		Lower	Upper
Paternal attitude toward TV viewing	0.19	-0.38	0.76	-0.01	-0.78	0.76	-0.49	-1.43	0.46	1.27	-1.04	3.58
Maternal attitude toward TV viewing	-0.04	-0.61	0.54	0.04	-0.73	0.81	-0.41	-1.29	0.47	-2.48	-5.36	0.40
Paternal attitude toward PA	0.17	-0.53	0.87	0.70	-0.26	1.66	-1.01	-2.08	0.06	-1.20	-4.76	2.37
Maternal attitude toward PA	0.00	-0.72	0.71	-0.30	-1.27	0.67	0.13	-0.95	1.20	0.59	-2.61	3.80
Paternal ST	**0.09** ^***^	**0.06**	**0.12**	0.10***	**0.06**	**0.14**	0.06*	0.01	0.11	0.07	-0.12	0.25
Maternal ST	**0.10** ^***^	**0.06**	**0.13**	0.07**	**0.03**	**0.12**	0.13***	0.07	0.19	0.38***	**0.21**	**0.55**
Paternal PA	0.07	-0.07	0.22	0.07	-0.11	0.26	0.06	-0.18	0.30	0.15	-0.57	0.88
Maternal PA	0.03	-0.12	0.17	0.11	-0.10	0.32	0.04	-0.21	0.29	-0.12	-0.87	0.63
Paternal cigarettes smoking	0.02	-0.02	0.07	0.03	-0.04	0.09	0.005	-0.07	0.08	0.06	-0.10	0.22
Maternal cigarettes smoking	0.03	-0.28	0.34	-0.40	-0.91	0.12	-30	-0.99	0.39	-0.80	-3.09	1.50
Paternal alcohol drinking	**0.30** ^*^	**0.05**	**0.56**	0.30	-0.05	0.65	-0.13	-0.55	0.28	0.14	-1.10	1.39
Maternal alcohol drinking	-0.14	-0.84	0.55	0.21	-0.74	1.16	-0.29	-1.49	0.91	-1.65	-4.78	1.48
Paternal attitude toward TV viewing × girls				0.40	-0.65	1.44						
Maternal attitude toward TV viewing × girls				-0.15	-1.18	0.89						
Paternal attitude towards PA × girls				-1.19	-2.55	0.16						
Maternal attitude towards PA × girls				0.65	-0.67	1.97						
Paternal ST × girls				-0.02	-0.08	0.04						
Maternal ST × girls				0.04	-0.02	0.11						
Paternal PA × girls				-0.01	-0.29	0.27						
Maternal PA × girls				-0.17	-0.45	0.12						
Paternal smoking × girls				-0.01	-0.10	0.07						
Maternal smoking × girls				0.53*	**0.01**	**1.06**						
Paternal drinking × girls				-0.004	-0.49	0.49						
Maternal drinking × girls				-0.76	-2.07	0.54						
Paternal attitude toward TV viewing × age							0.06	-0.04	0.17			
Maternal attitude toward TV viewing × age							0.04	-0.06	0.14			
Paternal attitude towards PA × age							0.12*	**0.01**	**0.23**			
Maternal attitude towards PA × age							-0.02	-0.13	0.10			
Paternal ST × age							0.003	0.00	0.01			
Maternal ST × age							-0.004	-0.01	0.00			
Paternal PA × age							0.002	-0.02	0.02			
Maternal PA × age							-0.001	-0.02	0.02			
Paternal smoking × age							0.002	0.00	0.01			
Maternal smoking × age							0.02	-0.02	0.06			
Paternal drinking × age							0.04	0.00	0.08			
Maternal drinking × age							0.02	-0.08	0.12			
Paternal attitude toward TV viewing × log(family income)										-0.13	-0.38	0.13
Maternal attitude toward TV viewing × log(family income)										0.27	-0.04	0.58
Paternal attitude toward PA × log(family income)										0.14	-0.24	0.53
Maternal attitude toward PA × log(family income)										-0.06	-0.41	0.28
Paternal ST × log(family income)										0.00	-0.02	0.02
Maternal ST × log(family income)										-0.03***	**-0.05**	**-0.01**
Paternal PA × log(family income)										-0.01	-0.09	0.07
Maternal PA × log(family income)										0.02	-0.07	0.10
Paternal smoking × log(family income)										0.00	-0.02	0.01
Maternal smoking × log(family income)										0.09	-0.17	0.35
Paternal drinking × log(family income)										0.02	-0.12	0.15
Maternal drinking × log(family income)										0.16	-0.18	0.50
Observations	4,648			4,648			4,648			4,648		
R^2^	0.18			0.19			0.18			0.18		
Adjusted R^2^	0.18			0.18			0.17			0.18		
F Statistic	32.24^***^			23.26^***^			23.83^**^			27.10^***^		

Significance: *p < 0.05; **p < 0.01; ***p < 0.001;

CI confidence interval, ST screen time, PA physical activity;

Adjustment for children’s age, gender, ethnicity, region, residence, parental education, parental employment status, and survey wave

### Moderation effects of children’s gender, age, and family income

As shown in Table 2, the positive interaction term of maternal smoking and children’s gender indicated that maternal smoking only tended to increase girls’ ST rather than boys’ ST (interaction term β = 0.53, 95% CI [0.01, 1.06], p < 0.05). In the second moderation model, a more positive attitude fathers had toward PA tended to decrease the ST of children (β = -1.01, 95% CI [-2.08, 0.06]) and this relationship was stronger for younger children (interaction term β = 0.12, 95% CI [0.01, 0.23], p < 0.05). In the last moderation effect model, the association between maternal ST and children’s ST was observed to decline as family income increased (interaction term β = -0.03, 95% CI [-0.05, -0.01], p < 0.001). Compared to children from high-income families, the ST of low-income family children was more strongly associated with their mothers’ ST.

### Moderation effect of the other parent’s attitudes and behaviors

Table 3 presents the results of how paternal and maternal influences on children’s ST were moderated by the other parent. Paternal ST and maternal ST occurring longer than 14 h/week were the only significant predictors of children’s ST (β = 0.06, 95% CI [0.00, 0.11], p < 0.05). Paternal ST was observed to have a larger positive association with children’s ST when mothers’ ST exceeded 14 h/week.

**Table 3:** Moderation effects of the other parent’s attitudes and health behaviors (N = 4,648)

	Estimate	95% CI		p-value	Model fit	
		Lower	Upper		Adjusted R^2^	*F*
**Maternal moderation effect on the impact of paternal attitudes and health behaviors on children’s ST**						
Paternal ST × Maternal ST exceeds 14 h/week	0.06*	**0.00**	**0.11**	**< 0.05**	0.18	30.05***
Paternal PA × Mother participates PA	-0.12	-0.39	0.15	0.38	0.18	30.43***
Paternal smoking × Mother ever smokes cigarettes	0.47	-0.15	1.09	0.13	0.18	30.44***
Paternal drinking × Mother frequently drinks alcohol	-0.87	-2.38	0.64	0.26	0.18	30.42***
Paternal TV preference × Mother has high TV watching preference	-0.30	-1.44	0.84	0.60	0.18	30.39***
Paternal attitude towards PA × Mother is conscious about PA	0.96	-0.32	2.23	0.14	0.18	30.45***
**Paternal moderation effect on the impact of maternal attitudes and health behaviors on children’s ST**						
Maternal ST × Paternal ST exceeds 14 h/week	0.03	-0.04	0.10	0.41	0.17	29.30***
Maternal PA × Father participates PA	0.10	-0.17	0.36	0.48	0.18	30.31***
Maternal smoking × Father ever smokes cigarettes	0.67	0.53	1.28	0.15	0.18	30.42***
Maternal drinking × Father frequently drinks alcohol	-0.40	-1.72	0.92	0.55	0.18	30.41***
Maternal TV preference × Father has high TV watching preference	-0.66	-1.81	0.50	0.27	0.18	30.40***
Maternal attitude towards PA × Father is conscious about PA	0.11	-1.18	1.40	0.87	0.18	30.36***

Significance: *p < 0.05; **p < 0.01; ***p < 0.001;

CI confidence interval, ST screen time, PA physical activity;

Adjustment for children’s age, gender, ethnicity, region, residence, parental education, parental employment status, and survey wave

### Lagged association between parental attitudes or behaviors and children’s ST

The results of the lagged associations are shown in Table 4. Paternal attitudes toward PA (β = -1.63, 95% CI [-3.12, -0.14], p < 0.05) and maternal cigarette smoking (β = 1.46, 95% CI [0.16, 2.76], p < 0.05) were associated with children’s ST measured at four years later. However, parental ST did not appear to have any lagged associations with children’s ST at a later time point.

**Table 4:** A cross-lagged panel model of T0 (2011) parental attitudes and behaviors and T1 (2015) children’s ST (n = 1014)

	Dependent variable: T1 children’s ST			
	β	95% CI		p-value
		Lower	Upper	
T0 Paternal attitude toward TV viewing	0.96	-2.23	4.15	0.56
T0 Maternal attitude toward TV viewing	0.36	-1.09	1.81	0.62
T0 Paternal attitude towards PA	**-1.63**	**-3.12**	**-0.14**	**< 0.05**
T0 Maternal attitude towards PA	-0.75	-2.47	0.96	0.39
T0 Paternal ST	-0.14	-0.49	0.22	0.45
T0 Maternal ST	0.10	-1.67	1.88	0.91
T0 Paternal PA	-0.23	-0.77	0.31	0.4
T0 Maternal PA	0.46	-0.03	0.95	0.06
T0 Paternal cigarette smoking	-0.02	-0.20	0.17	0.86
T0 Maternal cigarettes smoking	**1.46**	**0.16**	**2.76**	**< 0.05**
T0 Paternal alcohol drinking	0.47	-0.61	1.54	0.39
T0 Maternal alcohol drinking	-0.86	-2.30	0.58	0.24

Fit indices: χ^2^ = 202.15 (28); CFI = 0.98; NFI = 0.98; SRMR = 0.04; RMSEA = 0.07;

CI confidence interval, ST screen time, PA physical activity;

Adjustment for children’s age, gender, ethnicity, region, residence, parental education, parental employment status, and survey wave

### Sensitivity analysis

A sensitivity analysis was conducted to examine the robustness of the results. Parental age is an important factor that might affect their attitudes and behaviors. Nonetheless, parental age was not included in the main analyses as a control variable because it contained too many missing values. Therefore, we used the mean imputation method to fill in missing data on parental age in both waves. As shown in Supplementary Appendix II, results of the regression and CLPM based on the imputed dataset showed no apparent difference from the main analyses.

## Discussion

By utilizing a nationally representative sample of Chinese mother-father-child triads from the CHNS, this study explored the associations of parental attitudes and health behaviors with children’s ST over four years. Instead of well-studied parental attitudes about children’s screen use, the current study focused on parental attitudes toward their own health behaviors. Although we failed to identify significant associations between parental attitudes toward TV viewing and PA with children’s ST in the pooled OLS models, it is noteworthy that positive parental attitudes toward their own PA tended to decrease children’s ST four years later in the CLPM. The results of a recent study by Lucas et al. [10] extended the social determination theory [25] from the intrapersonal to the interpersonal level, suggesting the importance of parental intrinsic motivation for driving changes in children’s health behaviors. Similarly, this study highlights the concept that the more parents care about their engagement in PA, the less their children will use screens at later times.

This study also examined the associations of both screen use and nonscreen-related health behaviors of parents with children’s ST. Consistent with the results of previous studies [11, 26], we also found a significant positive association between parental ST and children’s ST, thus suggesting the existence of parental role modeling in shaping children’s screen use behaviors. This finding is in line with social cognitive theory, which posits that children’s behaviors are acquired from the environment by observing and imitating individuals around them [27]. A novel finding of the current study was the association between parents’ nonscreen-related health behaviors and children’s ST. With the pooled OLS model, we found a significant relationship between paternal alcohol drinking and children’s ST. Moreover, in the CLPM, maternal cigarette smoking was positively associated with children’s ST four years later. A previous study by Garriguet, Colley, and Bushnik [16] explored the associations between parents’ non-PA-related health behaviors, such as dietary patterns and substance use, and children’s PA but failed to identify any correlation. In addition to a different outcome variable, the difference in the findings between their study and our study might involve the cross-sectional design employed in their study, thus ignoring the potential lagged effects. In fact, strong evidence has proven the importance of parents as being both “gatekeepers” and “role models” in shaping children’s screen use behaviors [26, 28]. Parents’ nonscreen-related behaviors may not impact children’s ST as directly as parents’ screen-related parenting practices, such as rule setting (gatekeepers) or parental screen use (role models), but they are expressions of the household’s shared value toward healthy living [16]. The current study highlights that, as an important component of the family health climate, parental behaviors of “being themselves” may have a lagged effect on children’s lifestyles.

The current study also examined the parent–child dyadic relationship in the context of the other parent and found a larger positive father-children association in ST when mothers had longer ST. This finding demonstrated that synergistic negative effects of paternal and maternal ST may exist, which can increase children’s ST. Due to the fact that mothers were assumed to be the main caregivers for children, most of the early research on parental influence on children’s ST primarily focused on mother-child associations [29, 30]. An increase in female participation in the labor market has gradually altered the ideology about the role of mothers and fathers and has highlighted the importance of investigating the parent–child relationship from a family system perspective [17, 18]. The current study is among the first studies that explored the parental influences on children’s ST with a consideration of family members’ interrelationships and found a maternal moderating effect on the father-child dyadic relationship.

Furthermore, the current study examined whether parental influences are moderated by the gender, age, and family income of children. First, maternal smoking was more associated with girls’ ST than boys’ ST. This gender-specific relationship was in line with the previous finding of a more significant mother-daughter correlation in screen use behaviors [19]. Social cognitive theorists have pointed out that children observe and learn behaviors from role models of both genders, but they might tend to copy the behaviors of same-gender models [31]. Second, the current study showed that children’s age failed to moderate parental influences in most cases. Although some scholars have argued that the effect of parental role modeling would diminish as children grow older because children become more influenced by other role models, such as teachers or peers [32], the results of this study suggest that parents may continue to have an important influence on children’s ST. Third, this study identified children from low-income families as being more inclined to be impacted by maternal ST. This may be explained by the impact of household income on the gendered division of labor in households. As high-income families are able to afford paid childcare, mothers in low-income families may take on more responsibilities as caregivers [33]. Therefore, children from low-income families may spend more time with their mothers and be more influenced by their mothers’ health behaviors.

### Limitations

First, all of the health behaviors were measured in the CHNS by using self-report (6 years and older) or proxy-report (0- to 5- years old) questionnaires, which might introduce information bias. Other measurements for people’s health behaviors are suggested for future usage to diminish this bias, such as the 24-Hour Diary and Ecological Momentary Assessment (EMA) [34, 35]. Additionally, parental attitudes toward TV viewing were the only measurement of their attitudes toward screen use. As adults spend time on other screen devices, such as computers and smartphones, this one-item measurement might be insufficient. Future studies are suggested to measure parental attitudes toward different screen use behaviors, such as watching short videos or online chatting via smartphones or tablets. Moreover, the family health climate was not directly measured in CHNS. Future research could consider the use of validated scales, such as the Family Health Climate Scale (FHC-scale) [7]. Second, a previous intrapersonal-level study revealed that the synergy of unhealthy behaviors may have a more detrimental impact on one’s health than a simple cumulation of individual effects [15]. Although this study explored the influences of both screen use and nonscreen-related behaviors of parents on children’s ST, we did not examine the synergy of parental behaviors. Future studies could further utilize cluster analysis techniques, such as latent class analysis (LCA), to explore the synergistic effect of parental factors. Third, the current study only established the associations between parental factors and children’s ST, whereas the underlying mechanisms were not sufficiently explored, which should be further studied in the future. Fourth, the data used in the current study were collected in 2015 and earlier, when the use of new screen technologies such as mobile phones was not as prevalent as nowadays. The generalization of results therefore must be made cautiously. Last, even though the CHNS data is of national representativeness in China, the generalization of findings of the current study to other countries or cultural backgrounds should be cautious. For example, around 65% of Chinese parents had zero participation in PA in our sample, which might also be a cause of the insignificance between parental PA and children’s ST in the current study. Previous research has indicated that the PA level in adults might be higher in the Eastern Mediterranean, the Americas, and Europe than in Southern and Eastern Asia possibly due to the lack of sports centers and clubs in the latter regions [36]. We suggest future studies on people’s lifestyles pay more attention to cross-country differences.

## Conclusions and implications

Children establish a healthy lifestyle within the family system. This study included a set of less-explored parental attitudinal and behavioral factors affecting children’s ST and found that nonscreen-related parental attitudes and behaviors were associated with children’s ST, which provided a fresh perspective in designing short-term and long-term family-based interventions targeting ST reduction. In the short term, parental role modeling is influential on children’s ST, and a simultaneous reduction of both parents’ ST should be necessary. In addition to the role model effects of both parents, increasing fathers’ awareness of the importance of PA to their health may be effective in decreasing children’s ST. Additionally, more attention should be given to children from low-income families, as they may be more vulnerable to parents’ unhealthy behaviors, thus leading to negative health consequences. In the long term, programs for reducing children’s ST should be built through an integrative approach and should aim at promoting a healthy family climate by targeting parental attitudes and multiple health behaviors, especially regarding parents’ smoking and drinking.

## Electronic supplementary material

Below is the link to the electronic supplementary material.

Supplementary Material 1

## Data Availability

Data available at: http://www.cpc.unc.edu/projects/china/.

## Abbreviations

CHNS: China Health and Nutrition Survey
CLPM: Cross-lagged panel model
PA: Physical activity
ST: Screen time
