# Supplementary material for: Associations of parental attitudes and health behaviors with children’s screen time over four years
Source: BMC Public Health. 2023 Feb 8;23:289. doi: 10.1186/s12889-023-15212-x (PMC9909857; doi:10.1186/s12889-023-15212-x)
Supplement: Supplementary file 1 — Supplementary Material 1 [file 12889_2023_15212_MOESM1_ESM.docx]

**Appendix I. Assumption diagnostics**

This study examined 4 assumptions for the pooled OLS regression model.

1. **Linearity**

The general linear model assumes linear relationships between its independent and dependent variables. As shown in the first column of Figure 1, the dependent variable (ttST_wt.c, children’s screen time) demonstrated linear relationships with independent variables (parental attitudes and behaviors).


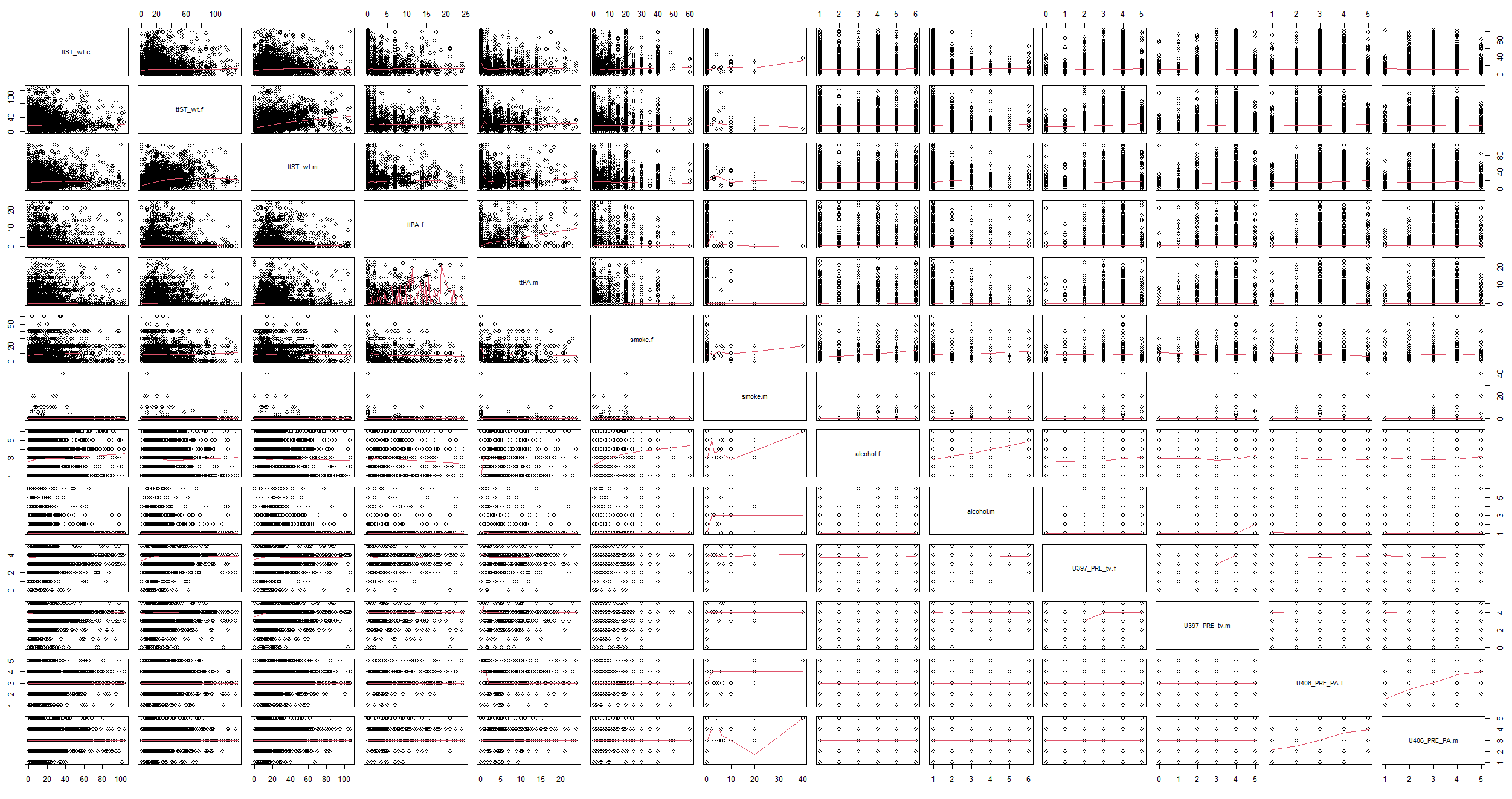


Figure 1. Linearity between the independent and dependent variables

From left to right: Children’s screen time; Paternal screen time; Maternal screen time; Paternal physical activity; Maternal physical activity; Paternal smoking; Maternal smoking; Paternal alcohol drinking; Maternal alcohol drinking; Paternal attitude toward TV viewing; Maternal attitude toward TV viewing; Paternal attitude toward physical activity; Maternal attitude toward physical activity

1. Homoscedasticity

To test whether variance of our model residuals is constant for each value of our independent variables, we produced scatter plots of the residual values against each independent variable. If there is no problem with heteroskedasticity, we will expect to see a random pattern of model residuals no matter what the value of our independent variable - we should not see any trend in the scatterplot and our regression and loess curves should follow a horizontal line.

We fitted a linear model by setting children’s screen time as the dependent variable, parental attitudes and health behaviors as independent variables, and children’s age, gender, ethnicity, residency, parental education, parental employment status as control variables. As shown in Figure 2, the residuals did not show any clear trend and the fitted loess curve was horizontal.


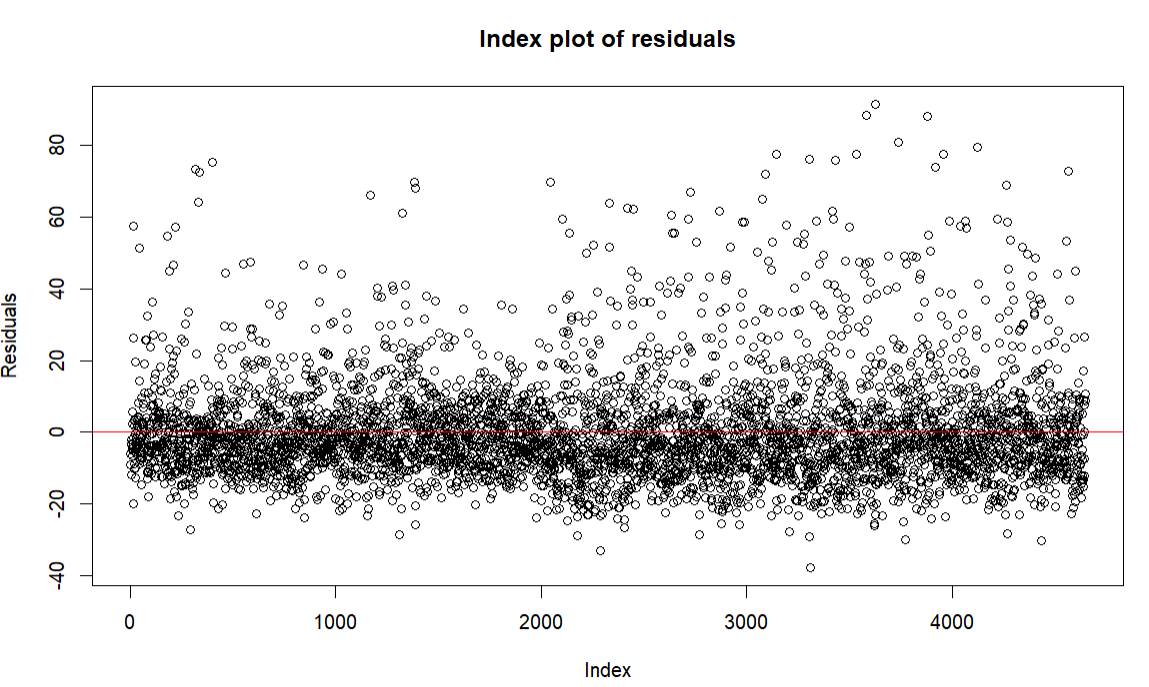


Figure 2. Plot of model residuals

1. Normality of Residuals

The histogram of model residuals showed that residuals were mostly normally distributed.


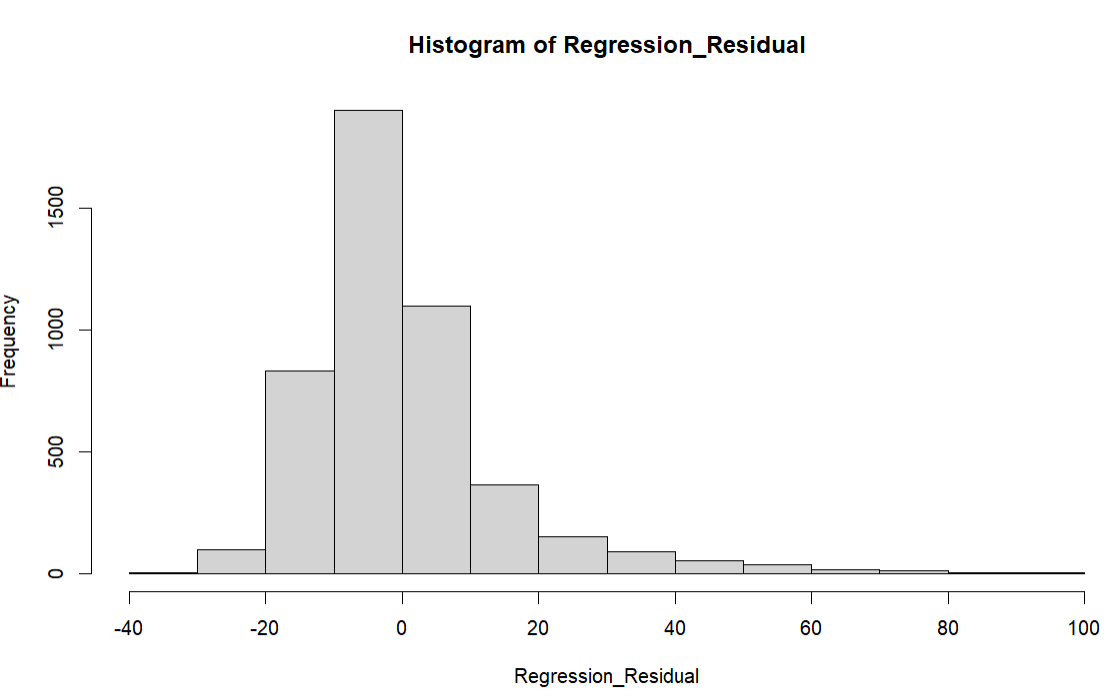


Figure 3. Histogram of model residuals

1. **No omitted variable**

We tested whether region and wave of survey were omitted variables in the regression using added variable plots. Firstly, we estimated the initial regression model (used in previous diagnostics) without the relevant variable and outputted a new data set with the residuals of that regression model. Secondly, we estimated a new model with region and wave of survey added and outputted a new data set with the residuals of the new model. Lastly, we plotted the two sets of residuals. As shown in Figure 4, the regression and loess lines are not horizontal. Thus, there is a reason to suspect that region and wave of survey are omitted variables. We included both of them in following regression analyses.


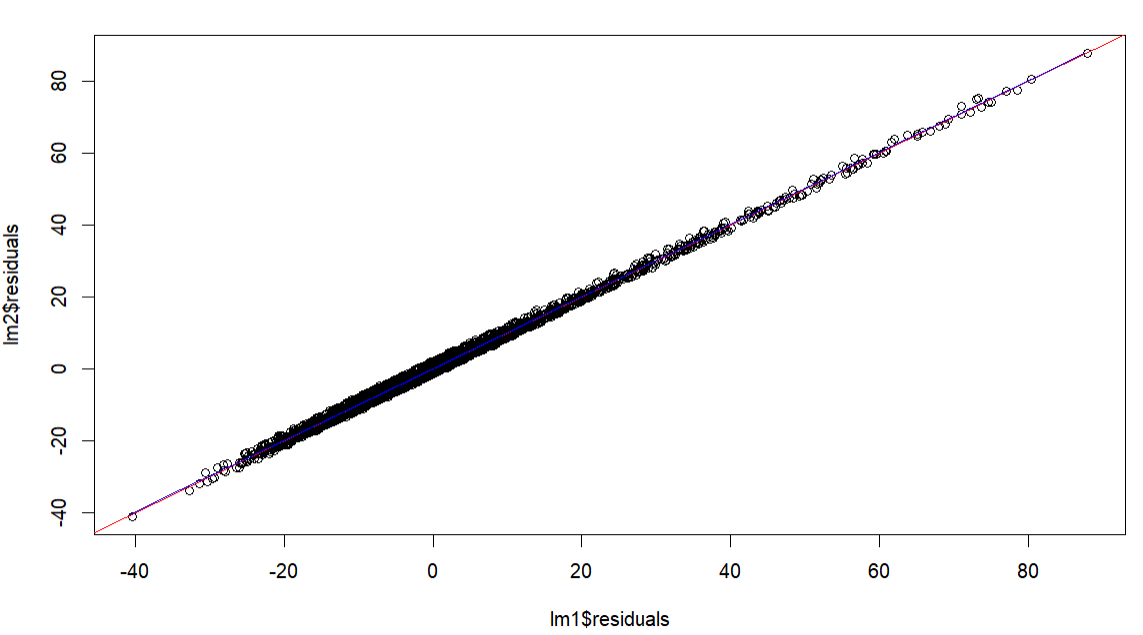


Figure 4. Added variable plot

**Appendix II. Sensitivity analysis**

**Table 1. Pooled OLS regression model of the effect of parental attitudes and health behaviors on child's ST with missing parental age imputed dataset**

|  | Dependent variable: Children's screen time | | | | | | | | | | | |
| --- | --- | --- | --- | --- | --- | --- | --- | --- | --- | --- | --- | --- |
|  | Base model | | | Children's gender moderation | | | Children's age moderation | | | Family income moderation | | |
|  | β | 95% CI | | β | 95% CI | | β | 95% CI | | β | 95% CI | |
|  |  | Lower | Upper |  | Lower | Upper |  | Lower | Upper |  | Lower | Upper |
| Paternal attitude toward TV viewing | 0.18 | -0.40 | 0.75 | -0.01 | -0.78 | 0.75 | -0.46 | -1.40 | 0.49 | 1.20 | -1.12 | 3.51 |
| Maternal attitude toward TV viewing | -0.03 | -0.61 | 0.55 | 0.05 | -0.72 | 0.82 | -0.31 | -1.19 | 0.56 | -2.44 | -5.31 | 0.43 |
| Paternal attitude toward PA | 0.17 | -0.53 | 0.88 | 0.70 | -0.26 | 1.65 | -0.93 | -2.00 | 0.14 | -1.15 | -4.75 | 2.44 |
| Maternal attitude toward PA | 0.01 | -0.70 | 0.73 | -0.28 | -1.26 | 0.69 | 0.19. | -0.88 | 1.25 | 0.57 | -2.67 | 3.80 |
| Paternal ST | **0.09***** | **0.06** | **0.12** | **0.10***** | **0.06** | **0.14** | **0.07**** | **0.02** | **0.11** | 0.07 | -0.12 | 0.25 |
| Maternal ST | **0.10***** | **0.06** | **0.13** | **0.08***** | **0.03** | **0.12** | **0.14***** | **0.08** | **0.19** | **0.38***** | **0.21** | **0.55** |
| Paternal PA | 0.07 | -0.07 | 0.22 | 0.07 | -0.12 | 0.26 | 0.06 | -0.18 | 0.29 | 0.14 | -0.58 | 0.86 |
| Maternal PA | 0.03 | -0.12 | 0.17 | 0.11 | -0.10 | 0.32 | 0.04 | -0.21 | 0.29 | -0.09 | -0.84 | 0.65 |
| Paternal cigarettes smoking | 0.02 | -0.02 | 0.07 | 0.03 | -0.04 | 0.09 | 0.00 | -0.07 | 0.07 | 0.07 | -0.09 | 0.23 |
| Maternal cigarettes smoking | 0.02 | -0.29 | 0.33 | -0.44 | -0.99 | 0.11 | -0.36 | -1.05 | 0.33 | -0.77 | -3.14 | 1.60 |
| Paternal alcohol drinking | **0.29*** | **0.04** | **0.55** | 0.29 | -0.06 | 0.64 | -0.14 | -0.55 | 0.28 | 0.16 | -1.08 | 1.40 |
| Maternal alcohol drinking | -0.16 | -0.86 | 0.54 | 0.20 | -0.76 | 1.16 | -0.22 | -1.44 | 0.99 | -1.68 | -4.77 | 1.40 |
| Paternal attitude toward TV viewing × girls |  |  |  | 0.37 | -0.67 | 1.41 |  |  |  |  |  |  |
| Maternal attitude toward TV viewing × girls |  |  |  | -0.14 | -1.18 | 0.90 |  |  |  |  |  |  |
| Paternal attitude towards PA × girls |  |  |  | -1.18 | -2.53 | 0.17 |  |  |  |  |  |  |
| Maternal attitude towards PA × girls |  |  |  | 0.65 | -0.67 | 1.97 |  |  |  |  |  |  |
| Paternal ST × girls |  |  |  | -0.02 | -0.08 | 0.04 |  |  |  |  |  |  |
| Maternal ST × girls |  |  |  | 0.05 | -0.02 | 0.11 |  |  |  |  |  |  |
| Paternal PA × girls |  |  |  | -0.01 | -0.29 | 0.26 |  |  |  |  |  |  |
| Maternal PA × girls |  |  |  | -0.16 | -0.44 | 0.12 |  |  |  |  |  |  |
| Paternal smoking × girls |  |  |  | -0.01 | -0.10 | 0.07 |  |  |  |  |  |  |
| Maternal smoking × girls |  |  |  | **0.58*** | **0.02** | **1.13** |  |  |  |  |  |  |
| Paternal drinking × girls |  |  |  | 0.002 | -0.49 | 0.49 |  |  |  |  |  |  |
| Maternal drinking × girls |  |  |  | -0.76 | -2.07 | 0.55 |  |  |  |  |  |  |
| Paternal attitude toward TV viewing × age |  |  |  |  |  |  | 0.06 | -0.05 | 0.16 |  |  |  |
| Maternal attitude toward TV viewing × age |  |  |  |  |  |  | 0.03 | -0.07 | 0.13 |  |  |  |
| Paternal attitude towards PA × age |  |  |  |  |  |  | 0.11 | 0.00 | 0.23 |  |  |  |
| Maternal attitude towards PA × age |  |  |  |  |  |  | -0.02 | -0.14 | 0.10 |  |  |  |
| Paternal ST × age |  |  |  |  |  |  | 0.00 | 0.00 | 0.01 |  |  |  |
| Maternal ST × age |  |  |  |  |  |  | 0.00 | -0.01 | 0.00 |  |  |  |
| Paternal PA × age |  |  |  |  |  |  | 0.00 | -0.02 | 0.02 |  |  |  |
| Maternal PA × age |  |  |  |  |  |  | 0.00 | -0.02 | 0.02 |  |  |  |
| Paternal smoking × age |  |  |  |  |  |  | 0.00 | 0.00 | 0.01 |  |  |  |
| Maternal smoking × age |  |  |  |  |  |  | 0.02 | -0.02 | 0.06 |  |  |  |
| Paternal drinking × age |  |  |  |  |  |  | 0.04 | 0.00 | 0.08 |  |  |  |
| Maternal drinking × age |  |  |  |  |  |  | 0.01 | -0.09 | 0.11 |  |  |  |
| Paternal attitude toward TV viewing × log(family income) |  |  |  |  |  |  |  |  |  | -0.12 | -0.37 | 0.13 |
| Maternal attitude toward TV viewing × log(family income) |  |  |  |  |  |  |  |  |  | 0.27 | -0.05 | 0.58 |
| Paternal atitude towards PA × log(family income) |  |  |  |  |  |  |  |  |  | 0.14 | -0.25 | 0.53 |
| Maternal atitude towards PA × log(family income) |  |  |  |  |  |  |  |  |  | -0.06 | -0.41 | 0.29 |
| Paternal ST × log(family income) |  |  |  |  |  |  |  |  |  | 0.00 | -0.02 | 0.02 |
| Maternal ST × log(family income) |  |  |  |  |  |  |  |  |  | **-0.03***** | **-0.05** | **-0.01** |
| Paternal PA × log(family income) |  |  |  |  |  |  |  |  |  | -0.01 | -0.08 | 0.07 |
| Maternal PA × log(family income) |  |  |  |  |  |  |  |  |  | 0.01 | -0.07 | 0.09 |
| Paternal smoking × log(family income) |  |  |  |  |  |  |  |  |  | -0.01 | -0.02 | 0.01 |
| Maternal smoking × log(family income) |  |  |  |  |  |  |  |  |  | 0.09 | -0.18 | 0.35 |
| Paternal drinking × log(family income) |  |  |  |  |  |  |  |  |  | 0.01 | -0.12 | 0.15 |
| Maternal drinking × log(family income) |  |  |  |  |  |  |  |  |  | 0.17 | -0.17 | 0.50 |
| Observations | 4,648 | | | 4,648 | | | 4,648 | | | 4,648 | | |
| R^2^ | 0.18 | | | 0.19 | | | 0.18 | | | 0.19 | | |
| Adjusted R^2^ | 0.18 | | | 0.18 | | | 0.17 | | | 0.18 | | |
| F Statistic | 30.52** | | | 22.82** | | | 23.00*** | | | 22.69** | | |

Significance: *p<0.05; **p<0.01; ***p<0.001;

CI confidence interval, ST screen time, PA physical activity;
Adjustment for children's age, gender, ethnicity, region, residence, parental age, parental education, parental employment status, and survey wave.

**Table 2. Moderation effect of the other parent’s attitudes and behavior with parental age imputed dataset**

|  | β | 95% CI | | p-value | Model fit | |
| --- | --- | --- | --- | --- | --- | --- |
|  |  | Lower | Upper |  | Adjusted R^2^ | *F* |
| **Maternal moderation effect on the impact of paternal attitudes and health behaviors on children's ST** | | | | | | |
| Paternal ST × Maternal ST exceeds 14 hrs/week | **0.06*** | **0.00** | **0.11** | **<0.05** | 0.18 | 28.55^***^ |
| Paternal PA × Mother participates PA | -0.12 | -0.39 | 0.15 | 0.38 | 0.18 | 28.90^***^ |
| Paternal smoking × Mother ever smokes cigarettes | **0.48*** | **0.06** | **0.89** | **<0.05** | 0.18 | 28.92^***^ |
| Paternal drinking × Mother frequently drinks alcohol | -0.85 | -2.37 | 0.68 | 0.28 | 0.18 | 28.90^***^ |
| Paternal TV preference × Mother has high TV watching preference | -0.31 | -1.44 | 0.83 | 0.60 | 0.18 | 28.87^***^ |
| Paternal attitude towards PA × Mother is conscious about PA | 0.94 | -0.34 | 2.22 | 0.15 | 0.18 | 28.93^***^ |
|  |  |  |  |  |  |  |
| **Paternal moderation effect on the impact of maternal attitudes and health behaviors on children's ST** | | | | | | |
| Maternal ST × Paternal ST exceeds 14 hrs/week | 0.03 | -0.04 | 0.10 | 0.39 | 0.17 | 27.81^***^ |
| Maternal PA × Father participates PA | 0.09 | -0.17 | 0.36 | 0.49 | 0.18 | 28.82^***^ |
| Maternal smoking × Father ever smokes cigarettes | **0.68***** | **0.37** | **0.99** | **<0.001** | 0.18 | 28.90^***^ |
| Maternal drinking × Father frequently drinks alcohol | -0.37 | -1.69 | 0.96 | 0.59 | 0.18 | 28.88^***^ |
| Maternal TV preference × Father has high TV watching preference | -0.65 | -1.81 | 0.51 | 0.27 | 0.18 | 28.88^***^ |
| Maternal attitude towards PA × Father is conscious about PA | 0.12 | -1.17 | 1.41 | 0.86 | 0.18 | 28.84^***^ |

Significance: *p<0.05; **p<0.01; ***p<0.001;

CI confidence interval, ST screen time, PA physical activity;
Adjustment for children's age, gender, ethnicity, region, residence, parental age, parental education, parental employment status, and survey wave.

**Table 3. Cross-lagged panel model of T1 parental attitudes and behaviors and T2 children's ST (n=1014) with parental age imputed dataset**

|  | **Dependent variable: T2 children's ST** | | | |
| --- | --- | --- | --- | --- |
|  | β | 95% CI | | P-value |
|  |  | Lower | Upper |  |
| T1 Paternal attitude toward TV viewing | 1.00 | -2.18 | 4.17 | 0.54 |
| T1 Maternal attitude toward TV viewing | 0.30 | -1.16 | 1.75 | 0.69 |
| T1 Paternal attitude towards PA | **-1.68** | **-3.17** | **-0.19** | **<0.05** |
| T1 Maternal attitude towards PA | -0.78 | -2.44 | 0.88 | 0.36 |
| T1 Paternal ST | -0.15 | -0.50 | 0.21 | 0.42 |
| T1 Maternal ST | 0.14 | -1.63 | 1.91 | 0.88 |
| T1 Paternal PA | -0.25 | -0.80 | 0.30 | 0.38 |
| T1 Maternal PA | 0.45 | -0.03 | 0.93 | 0.06 |
| T1 Paternal cigarette smoking | -0.02 | -0.18 | 0.15 | 0.87 |
| T1 Maternal cigarettes smoking | **1.49*** | **0.06** | **2.92** | **<0.05** |
| T1 Paternal alcohol drinking | 0.50 | -0.52 | 1.51 | 0.34 |
| T1 Maternal alcohol drinking | -0.84 | -2.29 | 0.61 | 0.26 |

Fit indices: χ^2^ = 203.1 (28); CFI = 0.98; NFI = 0.98; SRMR = 0.04; RMSEA = 0.08;

CI confidence interval, ST screen time, PA physical activity;
Adjustment for children's age, gender, ethnicity, region, residence, parental age, parental education, parental employment status, and survey wave.
